# A microRNA CRISPR screen reveals *microRNA-483-3p* as an apoptotic regulator in prostate cancer cells

**DOI:** 10.1038/s41419-025-08098-7

**Published:** 2025-10-24

**Authors:** Jonathan Tak-Sum Chow, Ayeisha Desjardins, Daniel K. C. Lee, Iulia A. Grigore, Linda Lee, Norman J. Fu, Stephanie Chau, Byeong Yeop Lee, Martino Marco Gabra, Leonardo Salmena

**Affiliations:** Department of Pharmacology and Toxicology, Faculty of Medicine, University of Toronto, Toronto, ON Canada

**Keywords:** miRNAs, Cancer genomics

## Abstract

The development of traditional protein-targeted cancer therapies is a slow and arduous process, often taking years or even decades. In contrast, RNA-based therapies targeting crucial microRNA (miRNA) offer a faster alternative due to the sequence-specific nature of miRNA inhibitor binding. This, combined with the capacity of individual miRNA to influence multiple cellular pathways, makes these small RNA attractive targets for cancer therapy. While miRNA are known to be dysregulated in prostate cancer (PCa), identifying their individual contributions to disease progression and the identification of therapeutically actionable miRNA targets in PCa has been challenging due to limited profiling and lack of screening tools. To address this need, we developed miRKOv2, a miRNA-only CRISPR knockout library enabling systematic, genome-wide loss-of-function screens to identify miRNA essential for PCa cell survival. Our screens uncovered 70 potential essential miRNA candidates, with miR-483 demonstrating the most significant impact on PCa cell viability. Functional characterization revealed that miR-483 disruption potentiated apoptosis in PCa cell lines. Mechanistically, we uncovered a novel regulatory axis wherein miR-483-3p directly modulates a BCLAF1/PUMA/BAK1 apoptotic signaling network, highlighting its critical role in maintaining PCa cell survival. Our findings provide novel insights into the complex regulatory role of miRNA in PCa progression and offer a potential therapeutic strategy for targeting miRNA-mediated pathways in metastatic disease.

## Introduction

Prostate cancer (PCa) is a prevalent solid malignancy, resulting in over 1.5 million new diagnoses and nearly 400,000 deaths each year around the world [1, 2]. The treatment paradigm for metastatic PCa has greatly improved over the last decade, owing to an improved understanding of androgen receptor signaling, and the development of targeted androgen therapies [2–4]. Despite advances in treatment that have improved survival, metastatic PCa remains an intractable and fatal disease, underscoring an urgent need for better therapies.

MicroRNA (miRNA) are small endogenous RNA, typically 19–25 nucleotides in length, that regulate gene expression post-transcriptionally. miRNA are crucial regulators of cellular homeostasis due to their capacity to control an estimated 60% of all protein-coding genes [5] and therefore almost every cellular process [6]. Consequently, dysregulation of miRNA is associated with many disease states, including cancer [7, 8]. The sequence-specific binding of miRNA inhibitors enables precise targeting, making miRNA viable drug targets. Furthermore, exogenous administration of miRNA can compensate for loss or mutation in disease, collectively positioning them as attractive therapeutic options for cancer [9].

PCa often exhibits dysregulation of miRNA processing, characterized by altered levels of DGCR8 and DICER [10–12]. Individual miRNA can also be disrupted, where gain or loss of expression is implicated in promoting metastatic PCa. For instance, enhanced miR-194-mediated repression of SOCS2 has been shown to drive PCa metastasis, while inhibition of miR-194 suppresses PCa cell invasion [13]. Inhibition of miR-221 and miR-222 resulted in the derepression of p27 and reduced the growth of in vivo xenografts [14]. Similarly, modified miR-21 inhibitors reduced tumor growth in vivo [15]. Together these examples underscore the therapeutic potential of miRNA-based therapies in cancer.

Despite the established link between miRNA dysregulation and PCa progression, identifying their individual contributions to disease progression has been challenging due to limited screening tools. Consequently, there is a need for the development of tools to functionally investigate miRNA roles in cancer. To address this gap, we developed a miRNA-focused CRISPR library called the **miR**NA-only **K**nock**O**ut (miRKO) version-2 (miRKOv2), a second iteration of the library that builds upon the original miRKO library [16].

To identify novel essential miRNA in PCa cells, we employed miRKOv2 to screen DU145 and LNCaP cells. We identified 70 candidate essential miRNA hits and further validated miR-483 as a putative essential miRNA in several PCa cell models through its regulation of apoptosis. We uncovered a novel regulatory axis where miR-483-3p directly regulates a BCLAF1/PUMA/BAK1 apoptotic signaling network. These findings provide evidence for miR-483-3p as a potential therapeutic target in metastatic PCa cells, where apoptotic evasion is a significant clinical challenge [17, 18].

## Results

### Generation of miRKOv2, and enhanced miRNA-only CRISPR-KO library

**Fig. 1:**
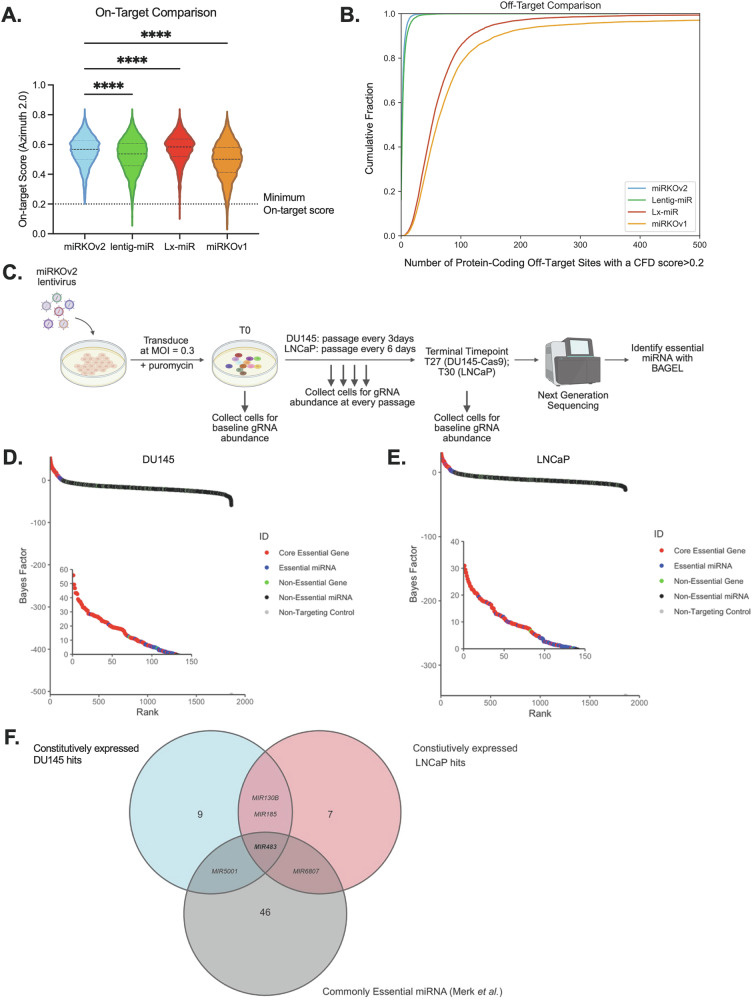
Identification of essential miRNA in PCa cells using a novel miRNA-focused CRISPR library. **A** On-target score comparison for gRNAs in the miRKOv2, lentiG-miR, Lx-miR and miRKOv1 libraries. Dotted line represents the recommended minimum on-target score of 0.2. Data represented by violin plots showing the first quartile, median and third quartile. ****p < 0.0001; One-way ANOVA with a Holm-Sidak’s multiple comparisons test. **B** Comparison of cumulative off-target loci with a CFD score > 0.2 for the miRKOv2, lentiG-miR, Lx-miR and miRKOv1 libraries. **C** Schematic representation of the miRKOv2 dropout screen in DU145-Cas9 and LNCaP cells. The screen was maintained at a 1000× gRNA representation and sequenced at a 500× gRNA representation. **D** Genes ranked hits by Bayes Factor (BF) at the T27 terminal timepoint for the DU145-Cas9 dropout screen. Hits with a BF > 0 and FDR < 0.1 were considered essential. Essential hits are shown in the inset. **E** Same as (**D**) but for the T30 terminal timepoint for the LNCaP dropout screen. **F** Overlap of constitutively expressed hits from the DU145-Cas9 and LNCaP screens and commonly essential genes curated by Merk et al$.$ [24] See also Fig. S1 and File S1.

To identify miRNA that are essential for PCa cell fitness we developed miRKOv2, a guide RNA library that incorporates the most updated on- and off-target gRNA scoring metrics [19]. miRKOv2 was designed to specifically target pre-miRNA hairpin stem regions to improve efficiency of miRNA disruption [20]. miRKOv2 contains positive control gRNA targeting validated core essential and non-essential protein coding genes [21–23]. We also included non-targeting gRNA as negative controls to make up 1% of the final library, to improve detection power. In sum, miRKOv2 consists of 6532 gRNA targeting 1649 pre-miRNA hairpins (3 or 4 gRNA per pre-miRNA), 800 gRNA targeting 100 core-essential and 100 non-essential genes (4 gRNA per gene), and 74 non-targeting gRNA. When benchmarked against other existing miRNA libraries [16, 24, 25] miRKOv2 exhibited comparable on-target and off-target profiles, however miRKOv2 strictly adheres to a 0.2 on-target score threshold, ensuring the exclusion of potentially inefficient gRNAs. This minimizes the risk of false negative results due to inefficient gRNA activity (Fig. 1A). Notably, miRKOv2 has the fewest off-target protein-coding loci with a CFD off-target score >0.2 which minimizes the likelihood of false positive results due to gRNA off-targets (Fig. 1B).

### Identification of essential miRNA in PCa cells

Dropout screens were performed in DU145 and LNCaP cells using miRKOv2 with day 27 and 30 days as the terminal time point, respectively (Fig. 1C). Using the Bayesian Analysis of EssentiaLity (BAGEL) tool, a supervised learning algorithm that employs Bayesian statistics [26], we identified 34 and 47 essential miRNA hits in the DU145 and LNCaP screens respectively, with 11 commonly identified in both screens for a total of 70 unique hits (Fig. 1D, E and S1A, B and File S1). Notably, many miRNA hits achieved BAGEL (Bayes Factor) scores that were comparable to core-essential coding gene controls, providing strong support for the notion that miRNAs can indeed function as crucial essential genes (Fig. 1D, E insets).

For miRNA candidate prioritization, we first assessed the expression level of candidate hits with miRNA expression profiles of a panel of PCa cell lines (DU145, PC3, 22Rv1, LNCaP and VCaP). We identified that 13 of 32 DU145 hits and 11 of 47 LNCaP hits were highly expressed (File S2). Furthermore, by integrating a list of common essential miRNA across several cancer types [24] we found *MIR483* to be commonly essential across multiple cancer types [24] (Fig. 1F and File S2). Given the limited understanding of miR-483 function in PCa, our study aimed to validate and elucidate a potential mechanism responsible for its essentiality.

### MIR483 is essential for PCa cell growth

**Fig. 2:**
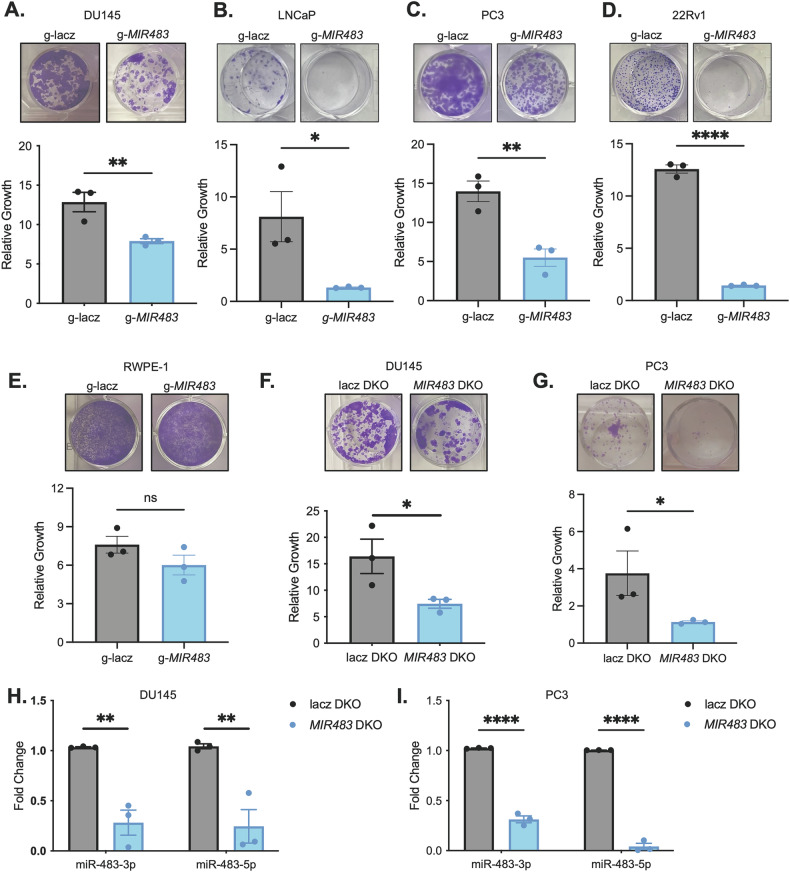
*MIR483* is essential for PCa cell growth. **A** Growth assays of miRKOv2-independent gRNA targeting *MIR483* in DU145-Cas9 cells. Representative images are shown on top and quantitation are shown on the bottom. **B** Same as (**A**) in LNCaP cells. **C** Same as (**A**) in PC3-Cas9 cells. **D** Same as (**A**) in 22Rv1 cells. **E** Same as (**A**) in RWPE-1 cells. **F** Growth assay of *MIR483* DKO in DU145-Cas9 cells. Representative images are shown on top and quantitation are shown on the bottom. **G** Same as (**F**) in PC3-Cas9 cells. **H** RT-qPCR of miR-483-3p and miR-483-5p following *MIR483* knockout with the DKO system in DU145-Cas9 cells. **I** Same as (**H**) in PC3-Cas9 cells. All data are represented as mean ± SEM from *n* = 3 independent experiments. *P* values obtained using an Unpaired one-tailed Student’s *t* test (**A–F**) or a Two-way ANOVA with a Holm-Sidak’s multiple comparisons test (**H**, **I**). *p < 0.05; **p < 0.01; ****p < 0.0001; ns not significant. See also Fig. S2 and File S2.

To validate *MIR483* as an essential gene, we assessed *MIR483*-targeting gRNA in the miRKOv2 screens to reveal that three of the four gRNA were depleted at the terminal timepoints in both screens (Fig. S2A, B). We also demonstrated that a *MIR483*-targeting gRNA distinct from those in miRKOv2 was able to reduce cell growth in DU145, LNCaP, 22Rv1 and PC3 cells (Fig. 2A-D). These data support that *MIR483* is an essential gene in PCa cells across diverse genetic backgrounds. Next, we observed that *MIR483* knockout in the premalignant immortalized prostate cell line RWPE-1 [27, 28] did not alter cell growth (Fig. 2E) suggesting that *MIR483* essentiality is restricted to malignant PCa cells.

To independently validate these findings, a dual gRNA knockout (DKO) strategy was employed (Fig. 2F, G). The DKO system was designed to generate two indels flanking the *MIR483* genomic locus, thereby completely excising the miRNA sequence (Fig. S2C). Using RT-qPCR, we confirmed the DKO-mediated disruption of mature miR-483-3p and miR-483-5p (Fig. 2H, I). Tracking of Indels by Deconvolution (TIDE) analysis [29] revealed a predominant 40–45 nucleotide deletion at the *MIR483* locus in both DU145 and PC3 cells (Fig. S2D, E), demonstrating successful DKO-mediated disruption. Furthermore, recognizing that *MIR483* resides within the second intron of *IGF2*, a gene encoding a peptide hormone essential for growth [30, 31], we confirmed that *MIR483* knockout did not interfere with IGF2 protein levels after *MIR483* knockout, ruling out *IGF2* disruption as a confounding factor in our experiments (Fig. S2F, G). Our findings demonstrate an essential role for *MIR483* in PCa cells, as confirmed by multiple experimental approaches.

### miR-483 targets apoptotic pathways and its knockout increases apoptosis in PCa cells

**Fig. 3:**
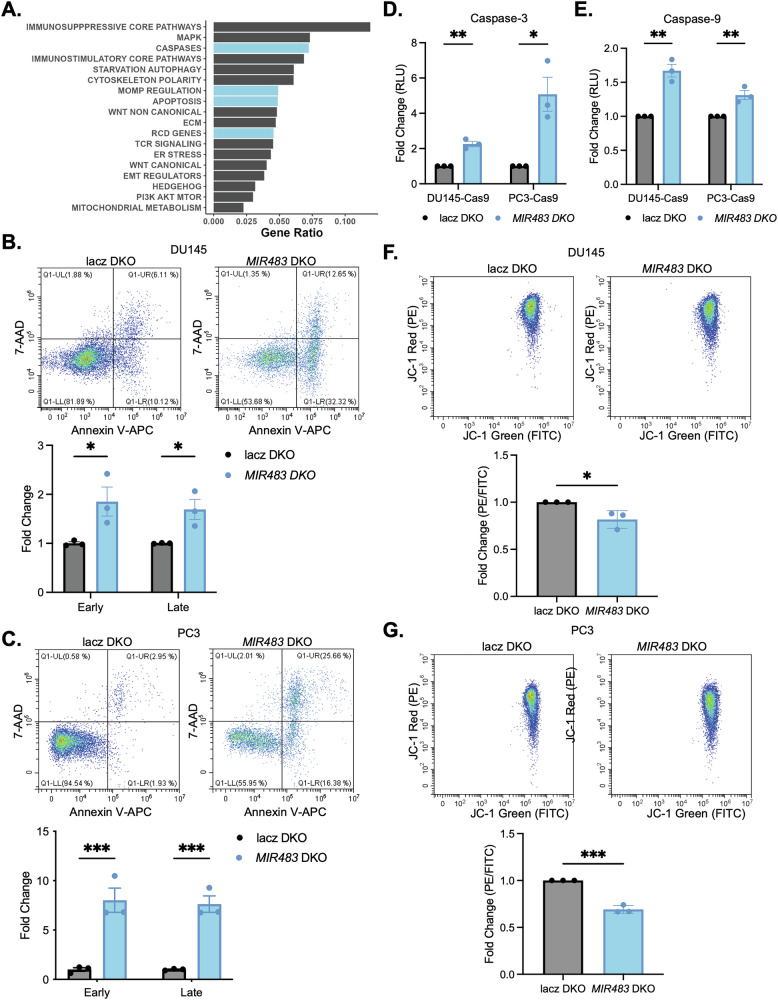
*MIR483* knockout increases apoptosis in PCa cells. **A** Pathway prediction analysis for miR-483-3p and miR-483-5p. **B** Annexin V/7-AAD flow cytometry of *MIR483* DKO in DU145-Cas9 cells. Representative plots are shown on top and quantitation is shown on the bottom. **C** Same as (**B**) in PC3-Cas9 cells. **D** Caspase-3 activity following *MIR483* DKO in DU145-Cas9 cells (left) and PC3-Cas9 cells (right). **E** Same as (**D**) with caspase-9 activity. **F** JC-1 flow cytometry following *MIR483* DKO in DU145-Cas9 cells. Representative plots are shown on the top and quantitation is shown on the bottom. **G** Same as (**F**) in PC3-Cas9 cells. All data represented as mean ± SEM from *n* = 3 independent experiments. *P* values obtained using a two-way ANOVA with a Holm-Sidak’s multiple comparisons test (**B, C**) or an Unpaired one-tailed Student’s *t* test (**D-G**). *p < 0.05; **p < 0.01; ***p < 0.001. See also Fig. S3.

The mechanisms underlying miR-483 essentiality were further explored using the Pathway Data Integration Portal (PathDIP) and Gene Set Enrichment Analysis (GSEA). PathDIP revealed an enrichment of apoptosis-related pathways downstream of miR-483 (Fig. 3A). Next, GSEA was performed on publicly available RNA-sequencing data from control and *MIR483* knockout cell lines to further investigate the gene expression changes associated with miR-483 alterations in diverse cell lines [24]. This analysis revealed that pathways associated with intrinsic apoptosis (as identified in the KEGG [32] and ACSN2 [33] databases) were enriched in *MIR483* knockout HT29, PC9, and MCF7 cells (Fig. S4). Interestingly, pathways associated with normal mitochondrial processes were enriched in control cells, suggesting that loss of miR-483 may disrupt these normal processes leading to intrinsic (mitochondrial) apoptosis (Fig. S4).

Reasoning that decreased cell growth in our KO models was a result of elevated apoptosis, we measured various apoptosis parameters. Firstly, *MIR483* DKO resulted in increased AnnexinV/7AAD staining in both DU145 and PC3 models (Fig. 3B, C). Secondly, we observed significant upregulation of caspase-3 and caspase-9 activity in both DKO models (Fig. 3D, E). Since caspase-9 is activated downstream of the apoptotic pathway [34], we also assessed activation of the intrinsic pathway using JC-1, a membrane-permeable cationic dye used to assess mitochondrial membrane potential (MOMP). *MIR483* DKO in DU145 and PC3 cells was accompanied by a significant decrease in MOMP (Figs. 3F, G and S3A,B). These data suggest that loss of miR-483 triggers MOMP and apoptosis. Together, these results support the notion that miR-483 controls apoptosis through the intrinsic pathway by regulating mitochondrial homeostasis.

### miR-483-3p targets BCLAF1/PUMA/BAK1 signaling to suppress intrinsic apoptosis

**Fig. 4:**
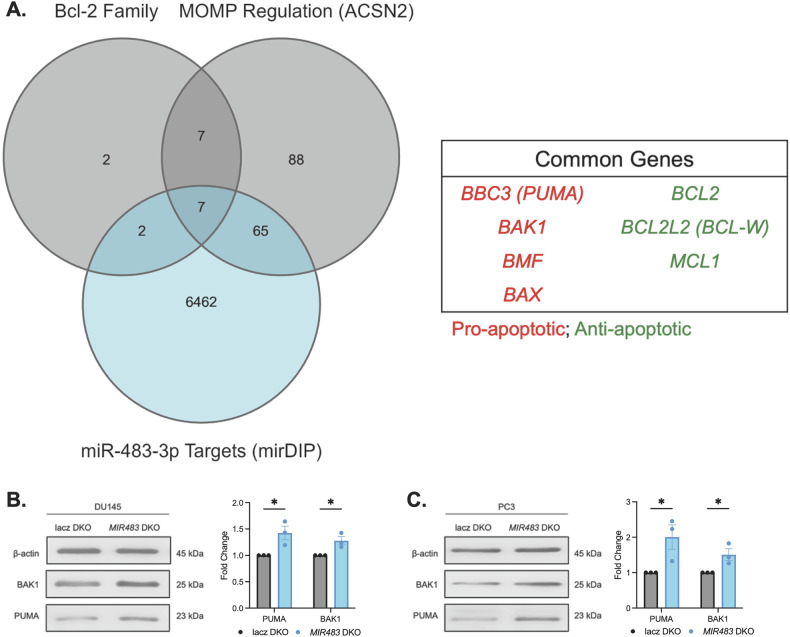
miR-483 is predicted to target apoptosis-related genes. **A** Overlap between predicted targets of miR-483-3p, MOMP-associated genes, and Bcl-2 Family genes (left). The seven common genes are listed on the right. **B** Western blot of PUMA and BAK1 in *MIR483* DKO DU145-Cas9 cells. Representative blot is shown on the left, and quantitation is shown on the right. **C** Same as (**B**) in PC3-Cas9 cells. All data represented as mean ± SEM from *n* = 3 independent experiments. *P* values obtained using an Unpaired one-tailed Student’s *t* test. * *p* < 0.05. See also Fig. S4 and Fig. S5.

Based on miR-483-5p and miR-483-3p expression in our PCa cell line panel (Fig. S5A) and in patient samples from the CPC-GENE dataset (Fig. S5B), we concluded that miR-483-3p is the dominant mature strand derived from the miR-483 pre-miRNA hairpin. Building upon reports in other malignancies demonstrating that miR-483-3p can suppress apoptosis [35–38], we aimed to elucidate its specific apoptotic role in PCa. By employing the miRNA Data Integration Portal (mirDIP) [39], we identified that 72 gene targets of miR-483-3p were associated with MOMP (Fig. 4A). Given the crucial role of the Bcl-2 family in regulating intrinsic apoptosis and MOMP [40, 41], we chose to specifically investigate miR-483-3p-targeted Bcl-2 family genes. Of the seven miR-483-3p-targeted Bcl-2 family genes, four are pro-apoptotic factors, supporting a potential role for miR-483-3p in suppressing apoptosis (Fig. 4A). Among these targets, PUMA and BAK1 emerged as strong candidates for being targeted by miR-483-3p in PCa cells. Experimental validation showed that PUMA, a well-known direct target of miR-483-3p in Wilms tumor and neuroblastoma [35, 37], was increased in *MIR483* DKO models (Fig. 4B, C). Similarly, BAK1 and BAX were also increased in *MIR483* DKO models (Figs. 4B, C and S5C). Notably, BAX protein was undetectable in DU145 cells (data not shown), consistent with previous reports [42], and BMF has limited pro-apoptotic activity [43], thus these genes were not examined further.

**Fig. 5:**
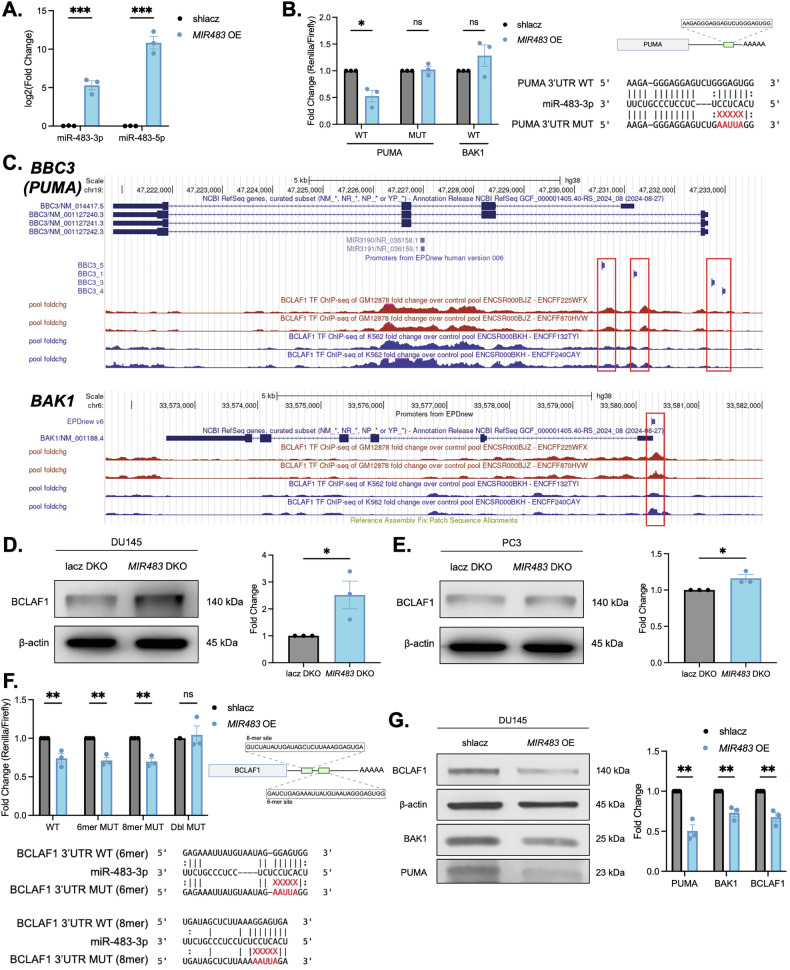
miR-483-3p suppresses a BCLAF1/PUMA/BAK1 signaling network. **A** RT-qPCR of miR-483-3p and miR-483-5p following *MIR483* overexpression in DU145 cells. **B** Dual luciferase assay of wild-type (WT) and seed-mutant (MUT) PUMA 3’UTR (left and middle), and WT BAK1 3’UTR reporters in DU145 cells. Schematic representation of the miR-483-3p binding site in the PUMA 3’UTR (top right) and the miR-483-3p seed mutation (bottom right). **C** Schematic representation of ENCODE ChIPseq peaks and the promoter regions of *BBC3* (top) and *BAK1* (bottom) from GM12878 cells (red track; GSE105550) and K562 cells (blue track; GSE105733). Alignment of ChIPseq peaks and promoter regions is highlighted in red. **D** Western blot of BCLAF1 in *MIR483* DKO DU145-Cas9 cells. Representative blot is shown on the left and quantitation is shown on the right. **E** Same as (**D**) in PC3-Cas9 cells. **F** Same as (**B**) with WT, 6-mer MUT, 8-mer MUT and double MUT BCLAF1 3’UTR reporter. Schematic representation of the miR-483-3p binding sites in the BCLAF1 3’UTR (top right) and the miR-483-3p seed mutations (bottom). **G** Western blot of BCLAF1, PUMA, and BAK1 in *MIR483* overexpressing DU145 cells. Representative blot is shown on the left and quantitation is shown on the right. All data represented as mean ± SEM from *n* = 3 independent experiments. *P* values obtained using an Unpaired one-tailed Student’s *t* test (**A, D, E, G**) or a two-way ANOVA with a Holm-Sidak’s multiple comparisons test (**B, F**). *p < 0.05; **p < 0.01; ***p < 0.001; ns, not significant.

Next, we aimed to confirm that PUMA and BAK1 were bona fide targets of miR-483-3p. First, we generated an overexpression construct for miR-483 by cloning its genomic locus containing pre-miR-483 plus 100 nucleotides of flanking downstream of a *U6* promoter in lentiMIR-puro. Overexpression of both miR-483-3p and miR-483-5p from this construct was confirmed by RT-qPCR (Fig. 5A). Next, the 3’UTR regions of *PUMA* and *BAK1* transcripts containing predicted binding sites were cloned into psiCHECK-2 and tested in a dual luciferase reporter assay. This assay validated that PUMA-3’UTR was a direct target of miR-483-3p, and was further confirmed with a seed-mutant construct which rescued targeting (Fig. 5B). Surprisingly, despite appearing to be de-repressed upon miR-483-3p knockout (Figs. 4B, C and S5C), direct targeting of BAK1 was not observed suggesting a more complex mechanism at play (Fig. 5B). We then hypothesized that BAK1 may be an indirect target of miR-483-3p, therefore we searched for other regulators of BAK1.

By leveraging publicly available transcription factor ChIP-seq data from the ENCODE project [44], we identified that *BAK1* and *BBC3* (encoding PUMA) are putative transcription targets of BCL2 Associated Transcription Factor 1 (BCLAF1) (Fig. 5C). Indeed, BCLAF1 has been reported to regulate several apoptotic genes and promote apoptosis in various cancers [45]. In support of this, we observed a significant enrichment of BCLAF1 transcriptional target genes following *MIR483* knockout in MCF7 and PC9 cells (Fig. S6). This finding provides compelling evidence that BCLAF1 may be associated miR-483-3p, potentially mediating some of the observed apoptotic effects. Upon examining BCLAF1 protein levels, we observed that it was de-repressed upon *MIR483* DKO in DU145 and PC3 cells (Fig. 5D, E). By examining the *BCLAF1* 3’UTR we identified two putative miR-483-3p binding sites, one with a 6-mer seed and a second with an 8-mer seed. We showed that miR-483 overexpression suppressed *BCLAF1* 3’UTR reporter activity, providing further evidence that BCLAF1 is a direct target of miR-483-3p. Peculiarly, single mutation of either the 6-mer seed or 8-mer seed sites was not sufficient to rescue the reporter repression, whereas mutation of both sites did rescue the suppression, confirming that BCLAF1 is a target of miR-483-3p (Fig. 5F). Supporting evidence of a role for miR-483-3p in regulating each of BCLAF1, PUMA and BAK1 was also observed upon miR-483 overexpression in DU145 cells (Fig. 5G), further indicating the existence of a miR-483-3p/BCLAF1/PUMA/BAK1 signaling network.

We sought to further validate that the regulation of this signaling network by miR-483-3p is mediated by the transcriptional activity of BCLAF1 by directly silencing *BCLAF1*. We observed that siRNA-mediated knockdown of *BCLAF1* resulted in downregulation of BAK1 protein levels, whereas PUMA protein levels remained unchanged in both DU145 and PC3 cells (Fig. S6B,C). These findings are in line with our observations that miR-483-3p can directly regulate PUMA independent of BCLAF1 whereas miR-483-3p indirectly regulates BAK1 through BCLAF1-mediated transcription. Altogether, we demonstrate the existence of this miR-483-3p/BCLAF1/PUMA/BAK1 signaling that underlies miR-483 essentiality in PCa cells.

### miR-483-3p inhibition increases docetaxel efficacy

**Fig. 6:**
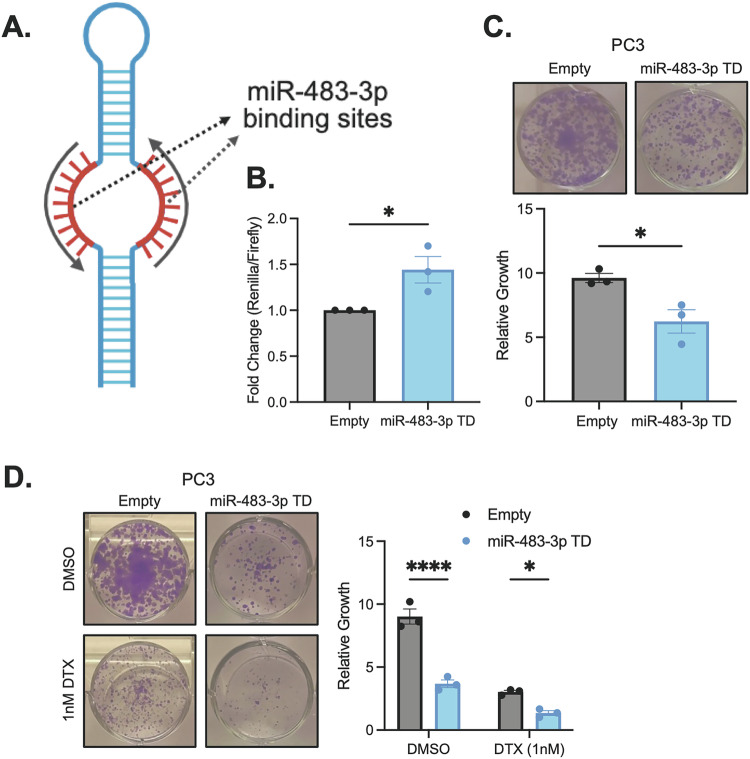
miR-483-3p is essential and sensitizes PCa cells to DTX. **A** Schematic representation of the miR-483-3p TD inhibitor. **B** Dual luciferase assay of miR-483-3p TD with a specific miR-483-3p sensor in PC3 cells. **C** Growth assay of miR-483-TD in PC3 cells. Representative images are shown on top and quantitation is shown on the bottom. **D** Growth assay of miR-483-3p TD in PC3 cells treated with either DMSO or 1 nM DTX. Representative images are shown on the left and quantitation is shown on the right. All data represented as mean ± SEM from *n* = 3 independent experiments. *P* values obtained using an Unpaired one-tailed Student’s *t* test (**B**, **C**) or a two-way ANOVA with a Holm-Sidak’s multiple comparisons test (**D**). *p < 0.05; ****p < 0.0001.

We next sought to validate that specific inhibition of miR-483-3p alone, rather than complete disruption of the miR-483 duplex with CRISPR, could confer essentiality phenotypes. For this, we employed Tough Decoys (TD), which are synthetic RNA molecules engineered to inhibit mature miRNA function by acting as high-affinity sponges or traps (Fig. 6A) [46, 47]. After confirming the efficacy of miR-483-3p TD using a specific miR-483-3p sensor (Fig. 6B), we also demonstrated that miR-483-3p TD resulted in a significant reduction in PC3 cell growth compared to TD controls (Fig. 6C). These data confirm that specific inhibition of miR-483-3p is essential for PCa cell fitness.

Next, the potential of miR-483-3p inhibition to enhance chemotherapeutic efficacy was explored by examining the consequences of combining miR-483-3p TD with docetaxel (DTX), a chemotherapeutic agent used for metastatic PCa [2–4]. Our results demonstrate that combined miR-483-3p inhibition with DTX significantly reduced PC3 cell growth compared to either treatment alone (Fig. 6D) suggesting that combining miR-483-3p inhibition with apoptosis-inducing chemotherapy, such as DTX, may offer improved treatment outcomes for metastatic PCa. We next investigated whether the improved DTX efficacy following miR-483-3p inhibition occurred synergistically. Accordingly, we performed synergy analysis with the SynergyFinder3 tool [48] using various doses of the miR-483-3p TD inhibitor and DTX concentrations, which revealed an overall additive interaction (synergy score = 3.966) between the two treatments under the Zero Interaction Potency model [49] (Fig. S7A). Intriguingly, despite overall additivity, we observed that specific dose combinations (e.g. 1 µg of miR-483-3p TD inhibitor and 0.1 nM DTX) did indeed achieve synergy (Fig. S7A). Similarly, we observed an decrease in the IC50 of DTX with an increasing amount of miR-483-3p TD inhibitor (Fig. S7B) further indicating that miR-483-3p inhibition improves DTX potency in PCa cells.

### miR-483 overexpression promotes growth in premalignant cells but not in PCa cells and attenuates docetaxel efficacy

**Fig. 7:**
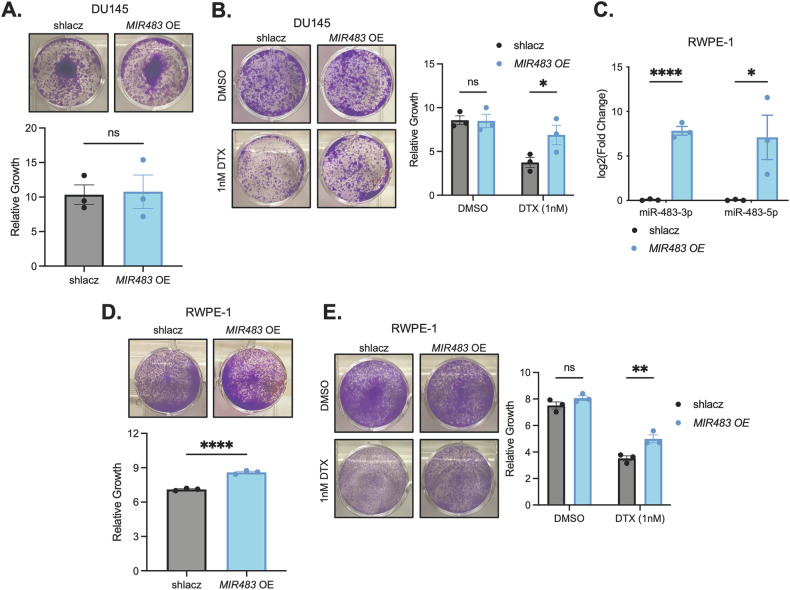
miR-483 promotes growth in premalignant cells but not PCa cells and attenuates DTX efficacy. **A** Growth assay of *MIR483* overexpression in DU145 cells. Representative images are shown on top and quantitation is shown on the bottom. **B** Growth assay of *MIR483* overexpression in DU145 cells treated with either DMSO or 1 nM DTX. Representative images are shown on the left and quantitation is shown on the right. **C** RT-qPCR of miR-483-3p and miR-483-5p following *MIR483* overexpression in RWPE-1 cells. **D** Same as (**A**) in RWPE-1 cells. **E** Same as (**B**) in RWPE-1 cells. All data represented as mean ± SEM from *n* = 3 independent experiments. *P* values obtained using an Unpaired one-tailed Student’s *t* test (**A**, **D**) or a two-way ANOVA with a Holm-Sidak’s multiple comparisons test (**B**, **C**, **E**). *p < 0.05; **p < 0.01; ****p < 0.0001; ns not significant.

To investigate the growth-promoting potential of miR-483 in PCa cells, we overexpressed miR-483 in DU145 cells. After confirming overexpression of both miR-483-3p and miR-483-5p mature miRNA strands (Fig. 5A), we failed to observe any growth difference (Fig. 7A). Surprisingly, miR-483-overexpressing cells were resistant to DTX cytotoxicity, suggesting that miR-483 may suppress apoptotic mechanisms normally engaged by DTX (Fig. 7B).

To further explore growth-promoting functions of miR-483, we overexpressed miR-483 in premalignant RWPE-1 cells (Fig. 7C). Unlike DU145 cells, we found that miR-483 overexpression promoted growth in RWPE-1 cells compared to control (Fig. 7D). Notably, miR-483 overexpression also conferred DTX resistance in RWPE-1 cells (Fig. 7E). Together, these results suggest that miR-483 can promote growth in non-transformed cells, however, PCa cells are insensitive to elevated miR-483 levels.

## Discussion

In this study, we generated miRKOv2: a novel CRISPR library targeting exclusively miRNA to screen PCa cell lines. Compared to previous iterations of miRNA KO libraries, miRKOv2 offers superior on-target efficacy, reduced off-target effects, and inclusion of positive control gRNA, permitting the use of the BAGEL algorithm for improved analysis of CRISPR dropout screens [26]. miRKOv2 is also compatible with other screen analysis algorithms, including MAGeCK [50] and casTLE [51], however, we recommend BAGEL which is superior [26]. We expect miRKOv2 to be a powerful resource to discover high-confidence miRNA candidates responsible for an array of phenotypes. miRKOv2 allowed us to identify essential roles for several miRNA in cellular growth and fitness, challenging the notion that miRNA function solely as fine-tuners of gene expression. Among the essential miRNA, our findings establish miR-483-3p as a master regulator of PCa cell survival, acting through direct modulation of the BCLAF1/PUMA/BAK1 apoptotic signaling network.

Previous studies have implicated miR-483-5p, the duplex partner of miR-483-3p, in promoting PCa growth. miR-483-5p overexpression was demonstrated to promote tumor growth in PCa cells by suppressing RBM5, a tumor suppressor gene that modulates apoptosis by regulating the alternative splicing of critical apoptotic mediators, including FAS and caspase-2 [52–54]. In a separate study, miR-483-5p was described as a crucial miRNA in a ceRNA network involving *LINC00908* and the tumor suppressor TSPYL5, which suppresses apoptosis to promote PCa progression [55]. Taken together with our findings described herein, it appears that both the 3p and 5p mature miRNA strands of miR-483 regulate key features of PCa cells involved in disease progression. In our study, unlike miR-483-5p, miR-483-3p was unable to promote PCa cell growth; however, its disruption was crucial for growth. In sum, miR-483-3p fits a gene profile characterized as essential for growth but does not demonstrate oncogenic features when overexpressed in PCa cells.

Nonetheless, its essentiality positions miR-483-3p as a putative therapeutic target in PCa. Notably, miR-483 disruption in non-malignant prostate epithelial cells did not result in any growth defect, a finding consistent with a previous study demonstrating that *MIR483* knockout mice developed normally and remained viable [56]. These data indicate the existence of a therapeutic window for targeting miR-483 in metastatic PCa, whilst sparing non-transformed tissues.

Based on the observed essentiality of miR-483 in PCa cell survival, we hypothesized that targeting miR-483-3p could render tumor cells more susceptible to apoptosis-inducing drugs. Upon examining the effect of combining miR-483-3p inhibition with docetaxel (DTX)—a standard therapy for metastatic PCa [2–4], we observed a significant additive effect. These findings suggest that the observed sensitization may be further enhanced through co-treatment with agents such as venetoclax, a Bcl-2 inhibitor currently undergoing clinical evaluation for metastatic PCa (NCT03751436) [57].

**Fig. 8:**
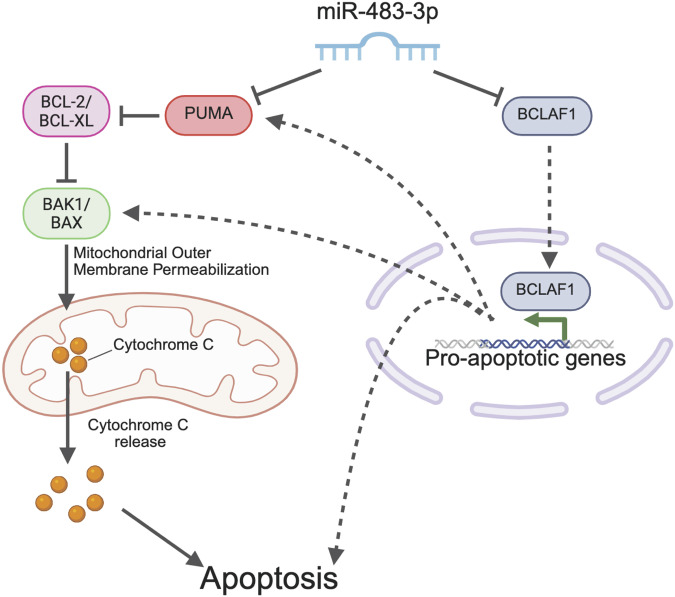
Schematic representation of the proposed miR-483-3p mechanism of essentiality. miR-483-3p is essential for PCa cell survival through suppression of apoptosis by targeting a BCLAF1/PUMA/BAK1 network. Inhibiting miR-483-3p in combination with apoptosis inducing agents can have additive effects at reducing PCa cell growth.

Bioinformatic analyses using publicly available datasets to identify candidate pathways associated with miR-483-mediated cell survival in PCa revealed a significant association between miR-483 and apoptosis-related genes, with Bcl-2 family members—PUMA, BMF, BAK1, and BAX—emerging as key potential mediators. Experimental validation in PCa cell lines demonstrated that disruption of miR-483 modulated the expression of PUMA and BAK1; however, only PUMA was confirmed as a direct target of miR-483-3p. Crucially, we discovered that BCLAF1, a regulator of both *PUMA* and *BAK1* expression, is also a direct miR-483-3p target [45]. This revealed a negative feed-forward loop, wherein miR-483-3p directly and indirectly suppresses Bcl-2 family genes via BCLAF1 (Fig. 8), enhancing its apoptotic regulatory capacity [8]. Our observation of a miR-483-3p/BCLAF1 feed-forward loop aligns with comparable regulatory networks in PCa, including miR-125b/p53, miR-17/92/MYC/E2F1, and miR-145/IGF1R, all described by Afshar et al. [58], which underscore the essential nature of miR-483 in PCa cells.

In sum, our study addresses the critical need for robust, large-scale methodologies to investigate miRNA function, particularly in metastatic PCa, a disease with limited treatment options and significant miRNA involvement [59]. Specifically, we identified *MIR483* as a novel essential gene in PCa, acting through the BCLAF1/PUMA/BAK1 apoptotic network (Fig. 8). Considering its ability to selectively target PCa cells, enhance DTX sensitivity, and spare normal prostate epithelial cells, miR-483 presents a promising therapeutic target for metastatic PCa. Collectively, our data positions miR-483-3p as a worthy candidate for further therapeutic exploration.

## Materials and methods

### Cell lines and drug treatments

DU145, PC3, LNCaP-FGC (LNCaP), and 22Rv1 cells (ATCC) were cultured in RPMI-1640 medium (Wisent Inc., 350-000-CL) supplemented with 10% fetal bovine serum (FBS; Wisent Inc., 080150) and 1% penicillin/streptomycin (Wisent Inc., 450-201-EL). HEK293T cells (ATCC) were cultured in DMEM medium (Wisent Inc., 319-005-CL) supplemented with 10% FBS and 1% penicillin/streptomycin. RWPE-1 cells (ATCC) were cultured in K-SFM medium supplemented with bovine pituitary extract and recombinant epidermal growth factor (Gibco, 17005042). Cas9^+^ DU145 and Cas9^+^ PC3 cells were generated through viral transduction with lentiCas9-Blast lentivirus and used for all knockout experiments. All cells were maintained at 37 °C with 5% CO_2_. DU145, PC3, and RWPE-1 cells were dosed with 1 nM or indicated concentrations of docetaxel (CST, 9886S) for the appropriate assays.

### Plasmids and viral vectors

The miRKOv2 library was cloned into a modified version of the lentiGuide-puro vector (Addgene #52963) containing a turboGFP cassette (lentiGuide-puro-P2A-GFP). Constitutive Cas9^+^ cell lines were made using the lentiCas9-Blast vector (Addgene #52962). Single guide RNAs (sgRNAs) targeting *MIR483*, or a double knockout (DKO) system employing two *MIR483* flanking sgRNAs were cloned into the lentiGuide-puro vector or the lentiCRISPRv2 (Addgene #52961) vector and used for validation experiments. For overexpression studies, the *MIR483* genomic locus with 100 bp of surrounding context and slack negative control sequences was cloned into the lentiGuide-puro vector. Tough Decoy sequences were cloned into the pLKO.1-puro vector (Addgene #8453) downstream of a *U6* promoter sequence. A sequence of four tandem repeat miR-483-3p binding sites from LSB-hsa-miR-483-3p (Addgene #103550) and all 3’UTR sequences were cloned into the psiCHECK-2 vector (Promega, C8021). Lentivirus was made using the pMDG.2 (Addgene #12259) and psPAX2 (Addgene #12260) vectors.

### Plasmid cloning

#### Single gRNA cloning

DNA oligos for single gRNA were annealed and cloned into the lentiGuide-puro or lentiCRISPRv2 vectors as described in Shalem et al. [60]. Single gRNA sequences are listed in Table S2.

#### DKO system cloning

A sequence containing the DKO system (gRNA-tracrRNA-U6-gRNA) was synthesized with flanking BsmBI digestion sites (IDT) (Table S3). This sequence was cloned into the lentiGuide-puro vector, similar to the single gRNA cloning described above and Shalem et al. [60].

#### Generation of MIR483 overexpression constructs

The *MIR483* genomic locus with 100 nucleotides up- and downstream, followed by a *U6* poly(T) termination sequence, was synthesized with flanking BsmBI digestion sites (IDT) (Table S3) and cloned into the lentiGuide-puro vector (Addgene). A negative control, lacz-targeting shRNA sequence was also synthesized (IDT) (Table S3) and cloned in a similar manner. We term this overexpression system the lentiMIR-puro system.

#### Tough Decoy inhibitor cloning

The Tough Decoy sequence for miR-483-3p was designed as described in Yoo et al. [46] and synthesized (IDT) (Table S3). The synthesized sequence was then amplified using primers containing AgeI and EcoRI digestion sites listed in Table S1 and cloned into the pLKO.1 vector.

#### miR-483-3p sensor cloning

A sequence of four tandem repeat miR-483-3p binding sites was amplified from LSB-hsa-miR-483-3p (Addgene) using primers listed in Table S1. The amplified sequence was cloned into the psiCHECK-2 vector (Promega) through Gibson cloning with the NEBuilder HiFi DNA Assembly Master Mix (NEB, E261L).

### Lentiviral production and transduction

9 × 10^6^ HEK293T cells were seeded in 15 cm dishes and co-transfected with 50 µg plasmid of interest, 30 µg psPAX2, and 15 µg pMDG.2 using the calcium phosphate method. Virus was concentrated through ultracentrifugation at 22,000 rpm for 2.5 h, resuspended in 50 µL PBS (Wisent Inc., 311-010-CL) and stored at −80 °C for later use. Target cells were transduced with lentivirus and supplemented with 8 µg/mL protamine sulfate (MP Bioomedicals, 02194729-CF) for 48 h prior to a change of medium. 24 h later, cells were selected with puromycin (2 µg/mL for 2 days; BioShop, PUR333) or blasticidin (750 µg/mL for 7 days; BioShop, BLA477) depending on the vector.

### Plasmid transfection

3 × 10^5^ cells (DU145) or 6 × 10^5^ cells (RWPE-1) were seeded in six-well plates, or 1 × 10^5^ cells were seeded in 12-well plates (PC3, DU145) 24 h prior to transfection. All plasmids were transfected using the Lipofectamine 2000 reagent (Invitrogen, 11668019) according to the manufacturer’s protocol and in antibiotic-free medium. Transfected cells were collected for their appropriate assays 24 h post-transfection. For miR-483 overexpression, 2 µg (6-well) or 1 µg (12-well) of each plasmid was transfected. For dual-luciferase sensor constructs, 500 ng (12-well) of each plasmid was transfected. For synergy experiments, 0, 0.5, 1, or 2 µg of the miR-483-3p TD inhibitor plasmid was transfected into 12-well.

### siRNA transfection

7.5 × 10^4^ cells were reverse-transfected with 100 nM non-targeting or *BCLAF1* SMARTPool siGENOME siRNA (Dharmacon, D-001206-13-20 and M-020734-01-0005, respectively) using DharmaFECT-1 (DU145) or DharmaFECT-2 (PC3) (Dharmacon, T-2001-03 and T-2002-01, respectively) in 48-well plates according to the manufacturer’s protocol. Cells were harvested for protein extraction 96 h post-transfection.

### miRKOv2 design and cloning

#### In silico design

All annotated pre-miRNA and the corresponding sequences were obtained from miRBase v22.2 [61]. HGNC gene symbols for each pre-miRNA were used as input for the Broad GPP sgRNA Designer [19] (https://portals.broadinstitute.org/gpp/public/analysis-tools/sgrna-design) to identify all possible gRNA. Next, gRNA were excluded based on on-target score (Rule Set 2 score <0.2) and off-target score (CFD Match Bin I score >3) [19]. Lastly, gRNA targeting non-loop regions of the pre-miRNA sequence were prioritized for selection. For every pre-miRNA locus, where possible four gRNA from the remaining pool were selected with a minimum of three gRNA required. Pre-miRNA that did not satisfy this requirement were excluded as targets in the final library. Core-essential and non-essential genes were selected based on Bayes Factor across previous CRISPR-Cas9 screens [21–23], where 100 core-essential genes with the largest Bayes Factor were chosen and 100 non-essential genes with smallest Bayes Factor were chosen. gRNA targeting these core-essential and non-essential genes was identified as described above and selected based on the combined ranking generated by the sgRNA Designer, such that each gene was targeted by four gRNA. 74 gRNA (1% of the final library) were randomly selected from a pooled list of non-targeting gRNA from previously published libraries [19, 62, 63]. The final miRKOv2 library contains 6532 gRNA targeting 1649 pre-miRNA loci, 400 gRNA targeting 100 core-essential genes, 400 gRNA targeting 100 non-essential genes, and 74 non-targeting gRNA.

#### Benchmarking analysis

To benchmark the miRKOv2 library against existing libraries, we sought to compare the on-target and off-target score profiles. Both the miRKOv2 library and the lentiG-mir library [24] utilize the Rule Set 2/Azimuth 2.0 algorithm for on-target scoring and the Cutting Frequency Determination (CFD) algorithm for off-target scores [19]. Since the Lx-miR library [25] and the miRKO library [16] used older on-target and off-target algorithms, we calculated these scores using the Rule Set 2/Azimuth 2.0 and the CFD algorithms. For on-target benchmarking, the distribution of on-target scores was compared between libraries. For off-target benchmarking, the cumulative fraction of loci with a CFD score >0.2 in a protein-coding gene was compared between libraries.

#### miRKOv2 library amplification and cloning

Selected gRNA for the miRKOv2 library were synthesized as a 73mer pooled oligo array (Custom Array) and amplified using primers listed in Table S1. The amplified oligo pool was cloned into the lentiGuide-puro-P2A-GFP vector through Gibson cloning (QuantiBio, 95190). The resulting Gibson reaction product was transformed into 25 µL electrocompetent cells (Endura, 60242) according to the manufacturer’s protocol and plated onto 8 LB-agar plates in 245 mm^2^ BioAssay dishes (Corning, 431111). Plasmid DNA from the resulting colonies was extracted with the QIAGEN Plasmid Maxi Kit (Qiagen, 12163).

### miRKOv2 dropout screen

2.5 × 10^7^ DU145 or LNCaP cells were transduced with miRKOv2 lentivirus at an MOI of 0.3 and at a~1000X gRNA coverage (~7.5 × 10^6^ cells) and selected with puromycin as described above. After puromycin selection (T0), the remaining pool of cells was split into three technical replicates, each maintained at a 1000X gRNA coverage. Remaining cells were harvested for genomic DNA extraction. Each screen replicate was passaged every 3 days for a total of 27 days (T3–T27) or every 6 days for a total of 30 days (T6–T30) for DU145 and LNCaP, respectively. Remaining cells at each time point were collected for genomic DNA extraction. Each screen was performed in a single biological replicate with three technical replicates.

### Screen sequencing and analysis

Genomic DNA was extracted from collected cells at each time point using the QIAamp DNA Blood Midi Kit (Qiagen, 51185) according to the manufacturer’s protocol. Samples were submitted to the Princess Margaret Genomics Centre (PMGC, University Health Network, Toronto, Canada) for NGS library preparation and sequencing. Samples were sequenced at a depth corresponding to ~500X gRNA coverage. Read counts from all timepoints can be found in File S3 for the DU145-Cas9 dropout screen or in File S4 for the LNCaP dropout screen.

Demultiplexed and trimmed reads were aligned to the miRKOv2 library using Bowtie2 tool [64]. After alignment, read counts were calculated and essential miRNA were identified using the Bayesian Analysis of Gene Essentiality (BAGEL) algorithm, as described in Hart and Moffat [26] and the reference set of gRNA targeting core-essential genes (*n* = 400) and nonessential genes (*n* = 400) [21–23] that were included in the miRKOv2 library design and using a supervised iterative learning method (*n* = 1000 iterations). A gene was considered to be essential if it had a BF > 0 and an FDR < 0.1. The optimal time point for hit identification was determined based on performance-recall analysis of identifying the reference set of core-essential and non-essential genes (Fig. S1A,B).

### Growth assays

For 6-day growth assays, 1 × 10^3^ cells (PC3), 5 × 10^3^ cells (DU145) or 2 × 10^4^ cells (RWPE-1) were seeded in 12-well plates. For 10-day growth assays, 1 × 10^3^ cells (DU145), 1 × 10^4^ cells (LNCaP) or 5 × 10^3^ cells (22Rv1) were seeded in 12-well plates. 24 h later and at the terminal timpoint, cells were fixed with 10% formalin (SigmaAldrich, HT501128-4L) prior to staining with 0.05% crystal violet dissolved in 40% v/v methanol and destaining with water. Stained plates were imaged and dissolved with 10% acetic acid for quantitation of absorption at 595 nm using the SpectraMax M3, using the SoftMaxPro v6 software (Molecular Devices).

### Protein extraction and Western Blot

Whole cell protein lysates were extracted from cells using 1X RIPA buffer (CST, 9806) supplemented with protease inhibitors (Roche, 11836163001) and sonicated at 30% amplitude. Blots were imaged using the KwikQuant Ultra Digital-ECL Substrate (Kindle Biosciences, R1002) and the KwikQuant Imager. The following primary antibodies were used in this study: BCLAF1 (1:1000; Invitrogen, PA5-55686), β-actin (1:10,000; CST, 4967), PUMA (1:1000; CST 12450T), BAK1 (1:1000; CST, 12105S), BAX (1:1000; CST, 5023T), IGF2 (1:1000; Invitrogen, MA5-17096). All uncropped original blots can be found in the Supplemental Material.

### RNA extraction and RT-qPCR

Total cellular RNA was extracted using the RNeasy Mini kit (Qiagen, 74104). miRNA cDNA synthesis was performed using the TaqMan Advanced miRNA cDNA Synthesis kit (Applied Biosystems, A28007). Quantitative PCR was performed on cDNA using the TaqMan Fast Advanced Master Mix (Applied Biosystems, 4444557) with TaqMan Advanced miRNA assays (Applied Biosystems, A25576) and the QuantStudio 3 Real-Time PCR system (Applied Biosciences) using the QuantStudio Design and Analysis software v1.2 (Applied Biosciences). All Ct values were normalized to hsa-miR-103a-3p and using the ΔΔCt method. The following TaqMan assays were used in this study: hsa-miR-483-3p (Applied Biosystems, 478122_mir), hsa-miR-483-5p (Applied Biosystems, 478432_mir), hsa-miR-103a-3p (Applied Biosystems, 478453_mir).

### Characterization of indels

The *MIR483* locus with 300 nucleotides up- and downstream was amplified using primers listed in Table S1 from extracted genomic DNA and submitted to The Centre for Applied Genomics (TCAG, The Hospital for Sick Children, Toronto, Canada) for Sanger sequencing with a sequencing primer listed in Table S1. The control and mir-483 DKO sequencing chromatograms were then input into the Tracking of Indels by Deconvolution (TIDE) tool [29] (https://tide.nki.nl/) for indel deconvolution and identification.

### miRNA–target interaction analysis

#### Amplification of 3’UTR sequences and cloning into reporter constructs

3’UTR fragments (up to 1.5 kb) were amplified from genomic DNA extracted from PC3 cells using primers listed in Table S1 and cloned into the digested psiCHECK-2 vector (Promega) using Gibson cloning with the NEBuilder HiFi DNA Assembly Master Mix (NEB, E261L).

#### Generation of seed mutations

Predicted miRNA binding sites were obtained using the DIANA-micro-T (2023) tool [65]. The resulting binding sites were aligned with the cloned 3’UTR reporters. Mutations in the seed region from positions 3–7 (GGAGU>AAUUA) were generated by site-directed mutagenesis using mutagenic primers listed in Table S1 and the Q5 Site Directed Mutagenesis kit (NEB, E0554S).

#### Dual luciferase assay and analysis

Target cells were transfected with reporter constructs. 24 h post-transfection, cells were harvested to perform the dual luciferase assay with the Dual-Glo Luciferase Assay System (Promega, E2920) according to the manufacturer’s protocol in white bottom 96 well plates (Corning, 3917). Luminescence was measured using the SpectraMax M3 using the SoftMaxPro v6 software (Molecular Devices). The Renilla/Firefly ratio for each well was calculated, and the ratio fold change was calculated relative to the corresponding negative control samples.

### Flow cytometry and fluorescence imaging

#### Annexin V/7-AAD staining

1 × 10^5^ cells were resuspended in 1X Annexin V binding buffer (Invitrogen, 88-8007-74) prior to incubation with APC-conjugated Annexin V antibody (Invitrogen, 88-8007-74) and 7-AAD (CST, 47501S) for 10 min on ice in the dark. Cells were analyzed with the CytoFlex flow cytometer (Beckman Coulter) using the CytExpert software v4 (Beckman Coulter). At least 1 × 10^4^ single cells were collected per sample. Fluorescence measurements for Annexin V and 7-AAD were detected using the APC and PC5.5 channels, respectively. Early apoptosis was defined as Annexin V^+^/7-AAD^-^ cells, and late apoptosis was defined as Annexin V^+^/7-AAD^+^ cells.

#### JC-1 staining

JC-1 detection and analysis were performed as previously described [66]. Briefly, 1 × 10^5^ cells were stained with 1uM JC-1 (CST, 92891S) for 30 min at 37 °C in the dark prior to analysis by flow cytometry or fluorescence imaging. For flow cytometry, cells were washed with PBS (Wisent) prior to analysis with the CytoFlex flow cytometer (Beckman Coulter) using a 488 nm laser and the CytExpert software v4 (Beckman Coulter). At least 1 × 10^4^ single cells were collected per sample. Fluorescence measurements for JC-1 aggregates and JC-1 monomers were detected using the PC5.5 and FITC channels, respectively. For fluorescence imaging, cells were washed and imaged in PBS at 40× objective using the EVOS FL inverted fluorescence microscope. JC-1 aggregates and JC-1 monomers were imaged using the Texas-Red and GFP filters. Brightness for all images was adjusted to improve the fluorescence signal.

### Caspase activity assays

1 × 10^4^ cells were collected to assess caspase activity using the caspase-3 or caspase-9 glo reagents (Promega, G8090 and G8210, respectively) according to the manufacturer’s protocol in white bottom 96 well plates (Corning). The resulting luminescence was measured with the SpectraMax M3 using the SoftMaxPro v6 software (Molecular Devices).

### Synergy analysis

PC3 cells transfected with various amounts of the miR-483-3p TD inhibitor were plated for a 6-day growth assay as described above. Cells were dosed with DMSO or 0.1, 1, 10, or 100 nM of docetaxel. Cell viability was determined using the absorbance of crystal violet staining and normalized to empty vector-transfected and DMSO-treated cells. Synergy was quantified with the SynergyFinder3 tool (https://synergyfinder.fimm.fi/) [48] using the Zero Interaction Potency (ZIP) model, which integrates both the Bliss independence and Loewe additivity models [49]. The following synergy score thresholds were used as previously reported [67–69]: <0 denotes an antagonistic interaction; 0–5 denotes an additive interaction; and >5 denotes a synergistic interaction.

### miRNA expression sequencing and analysis

Total RNA from DU145, PC3, LNCaP, 22Rv1, and VCaP cells was extracted using the RNeasy Mini kit (Qiagen) prior to submission to the TCAG sequencing facility (The Hospital for Sick Children, Toronto, Canada) for small RNA library and sequencing on the Illumina NovaSeq 6000. The resultant sequence reads were demultiplexed and trimmed using Cutadapt [70]. Reads were then aligned and annotated to miRBase v22.2 [61] using the BWA alignment tool (v0.7.17) [71]. miRNA were considered to be constitutively expressed if they had ≥5 reads in at least 80% of the cell line panel. miRNA read counts can be found in File S5.

### miRNA expression analysis in patient samples

miRNA expression profiles from patients in the CPC-GENE patient dataset were obtained from GSE135535. The expression profiles of miR-483-3p and miR-483-5p were then extracted and plotted.

### Pathway analyses

#### Prediction of downstream miRNA targets and pathways

Predicted targets and pathways downstream of mir-483 mature strands were identified using the miDIP target prediction tool [39] and mirDIP-integrated pathDIP v.5 tool [72], respectively. For pathway predictions, the top 1% of predicted targets were selected for pathway enrichment analysis using the pathDIP algorithm [72]. The analysis was conducted using literature-curated pathway associations related to the Atlas of Cancer Signaling Network v2 (ACSN2) [33]. Significantly enriched pathways were identified based on a Bonferroni *q* < 0.05. The gene ratio was calculated based on the ratio between predicted targets and pathway gene size.

#### Gene set enrichment analysis (GSEA)

GSEA was performed on the GSE242259 (Merk et al. [24]) dataset using GSEA v.4.3.0 provided by the Broad Institute [73, 74] (https://www.gsea-msigdb.org/gsea/index.jsp). Samples were split by *MIR483* KO or control. Enriched gene sets were identified by 1000 gene set permutations. Gene sets with a nominal *p*-value < 0.05 were considered significantly enriched.

The curated KEGG (CP:KEGG) gene set was obtained from MSigDB Collections [32] (https://www.gsea-msigdb.org/gsea/msigdb/collections.jsp).

The curated ACSN2 gene set was obtained from the Atlas of Cancer Signaling Network [33] (https://acsn.curie.fr/ACSN2/downloads.html).

The BCLAF1 target gene set was obtained from Harmonizome 3.0 database [75] (https://maayanlab.cloud/Harmonizome/gene_set/BCLAF1/ENCODE+Transcription+Factor+Targets).

### Analysis of transcription factor chromatin immunoprecipitation sequencing (TF-ChIPseq) data

BCLAF1 TF-ChIPseq peaks in K562 cells (GSE105733) and GM12878 cells (GSE105550) were obtained from the ENCODE project [44]. Promoter regions were obtained and imported from the Eukaryotic Promoter Database (EPD) [76]. BCLAF1 TF-ChIPseq peaks and promoter regions for *BBC3 (PUMA)* and *BAK1* were aligned to the GRCh38 reference genome and visualized with the UCSC Genome Browser [77] (https://genome.ucsc.edu).

### Statistical analysis

All statistical analyses were conducted using GraphPad Prism v9. All data are from three independent experiments unless otherwise indicated. Where specified in the experimental section, an unpaired one-tailed Student’s *t* test was used for comparisons between two groups, and one-way ANOVA or two-way ANOVA tests were used for comparisons between more than two groups where appropriate. Multiple comparisons were calculated using the Holm-Sidak method. *, **, *** and **** denote *p* < 0.05, <0.01, <0.001 and <0.0001, respectively. n.s. denotes no significance.

## Supplementary information

Supplemental File S1

Supplemental File S2

Supplemental File S3

Supplemental File S4

Supplemental File S5

Supplementary Figure Legends

Supplemental Table S1

Supplemental Table S2

Supplemental Table S3

Supplemental Figure S1

Supplemental Figure S2

Supplemental Figure S3

Supplemental Figure S4

Supplemental Figure S5

Supplemental Figure S6

Supplemental Figure S7

Original Uncropped Western Blots

## Data Availability

Any additional information and materials in this paper are available from the corresponding author upon reasonable request.
