# Supplementary material for: A microRNA CRISPR screen reveals microRNA-483-3p as an apoptotic regulator in prostate cancer cells
Source: Cell Death Dis. 2025 Oct 24;16(1):752. doi: 10.1038/s41419-025-08098-7 (PMC12552650; doi:10.1038/s41419-025-08098-7)
Supplement: Supplementary file 6 — Supplementary Figure Legends [file 41419_2025_8098_MOESM6_ESM.docx]

**SUPPLEMENTAL MATERIAL LEGENDS**

**File S1. Excel file containing all Bayes Factor scores at the terminal timepoints for the DU145-Cas9 and LNCaP dropout screens. Related to Figure 1.**

**File S2. Excel file containing expression of the 32 miRNA hits from the DU145-Cas9 dropout screen and 47 miRNA hits from the LNCaP dropout screen in a PCa cell line panel. Related to Figure 2.**

**File S3. Read count table for every time point in the DU145-Cas9 dropout screen.**

**File S4. Read count table for every time point in the LNCaP dropout screen.**

**File S5. Read count table for the miRNA profiling of a PCa cell line panel.**

**File S6. Supplementary Material Legends and Supplementary Figure Legends.**

**Table S1. Excel file containing primer sequences used in this study.**

**Table S2. Excel file containing the single gRNA sequences used in this study.**

**Table S3. Excel file containing synthesized sequences used in this study.**

**SUPPLEMENTARY FIGURE LEGENDS**

**Figure S1. Quality control of the miRKOv2 dropout screen in DU145-Cas9 cells. Related to Figure 1. A.** Precision-Recall analysis of all time points in the DU145-Cas9 dropout screen. Area under the curve (AUC) calculations are shown in the inset. **B.** Same as (**A**) but for the LNCaP dropout screen.

**Figure S2. Validation of *MIR483* as an essential gene in PCa cells. Related to Figure 2. A.** Log_2_ transformed fold-changes of each gRNA from the miRKOv2 library across all timepoints in the DU145-Cas9 screen. **B.** Same as (**A**) but in the LNCaP dropout screen. **C.** Schematic representation of the DKO system where two gRNA target opposite ends of the *MIR483* gene. **D.** Indel characterization of the *MIR483* locus in *MIR483* DKO DU145-Cas9 cells. **E.** Same as (**D**) in PC3-Cas9. **F.** Western blot of IGF2 in *MIR483* DKO DU145-Cas9 cells. Representative blot is shown on the left and quantitation is shown on the right. **G.** Same as (**F**) in PC3-Cas9 cells. All data in (**F,G**) is represented as mean ± SEM from n=3 independent experiments. P-values obtained using an Unpaired one-tailed Student’s t-test. n.s. not significant.

**Figure S3. *MIR483* knockout induces mitochondrial permeabilization. Related to Figure 3. A.** Representative fluorescent images taken of JC-1 stained control and *MIR483* DKO DU145-Cas9 cells. Scale bar represents 100um. **B.** Same as (**A**) in PC3-Cas9 cells. Brightness for all images was adjusted to improve fluorescence signal.

**Figure S4. *MIR483* knockout is associated with apoptotic pathways and negatively associated with mitochondrial pathways. Related to Figure 4.** **A.** Gene set enrichment analysis of RNAseq of control and *MIR483* knockout MCF7. **B.** Same as (**A**) in PC9 cells. **C.** Same as (**A**) in HT29 cells. All data obtained from the GSE242259 dataset[(24)](https://sciwheel.com/work/citation?ids=16999546&pre=&suf=&sa=0).

**Figure S5. Expression of miR-483-3p in DU145 and PC3 cells. Related to Figure 4.** **A.** Log_2_ transformed normalized expression of miR-483-3p and miR-483-5p in a PCa cell line panel of LNCaP, VCaP, 22Rv1, DU145 and PC3 cells. **B.** Same as (**A**) in the CPC-GENE dataset (GSE135535). **C.** Western blot of BAX in *MIR483* DKO PC3-Cas9 cells. Representative blot is shown on the left and quantitation is shown on the right. All data is represented as mean ± SEM from n=3 independent experiments (**A, C**) or from n=320 patients (**B**). P-values obtained using an Unpaired one-tailed Student’s t-test. ** *p* < 0.01.

**Figure S6. BCLAF1 regulates BAK1 and PUMA downstream of miR-483-3p. Related to Figure 5.** **A.** Gene set enrichment analysis using a curated gene set of BCLAF1 targets of RNAseq of control and *MIR483* knockout MCF7 and PC9 cells. All data obtained from the GSE242259 dataset. **B.** Western blot of BCLAF1, BAK1 and PUMA following siRNA mediate knockdown of *BCLAF1* in DU145 cells. Representative blot is shown on the left and quantitation is shown on the right. **C.** Same as (**B**) in PC3 cells. All data in (**B,C**) All data is represented as mean ± SEM from n=3 independent experiments. P-values obtained using an Unpaired one-tailed Student’s t-test. * *p* < 0.05; ** *p* < 0.01; *** *p* < 0.001; ns, not significant.

**Figure S7. miR-483-3p inhibition is additive with DTX treatment. Related to Figure 6. A.** Synergy map of a 4x3 dose matrix of the miR-483-3p TD inhibitor and DTX, respectively, in PC3 cells. The predicted most synergistic area is shown with the shaded box. **B.** DTX dose response curves of various amounts of the miR-483-3p TD inhibitor transfected into PC3 cells. Log(IC50) values (nM) are shown in the table below. All data is represented as mean ± SEM from n=3 independent experiments.
