# Supplementary material for: A microRNA CRISPR screen reveals microRNA-483-3p as an apoptotic regulator in prostate cancer cells
Source: Cell Death Dis. 2025 Oct 24;16(1):752. doi: 10.1038/s41419-025-08098-7 (PMC12552650; doi:10.1038/s41419-025-08098-7)
Supplement: Supplementary file 17 — Original Uncropped Western Blots [file 41419_2025_8098_MOESM17_ESM.pptx]

## Slide 1
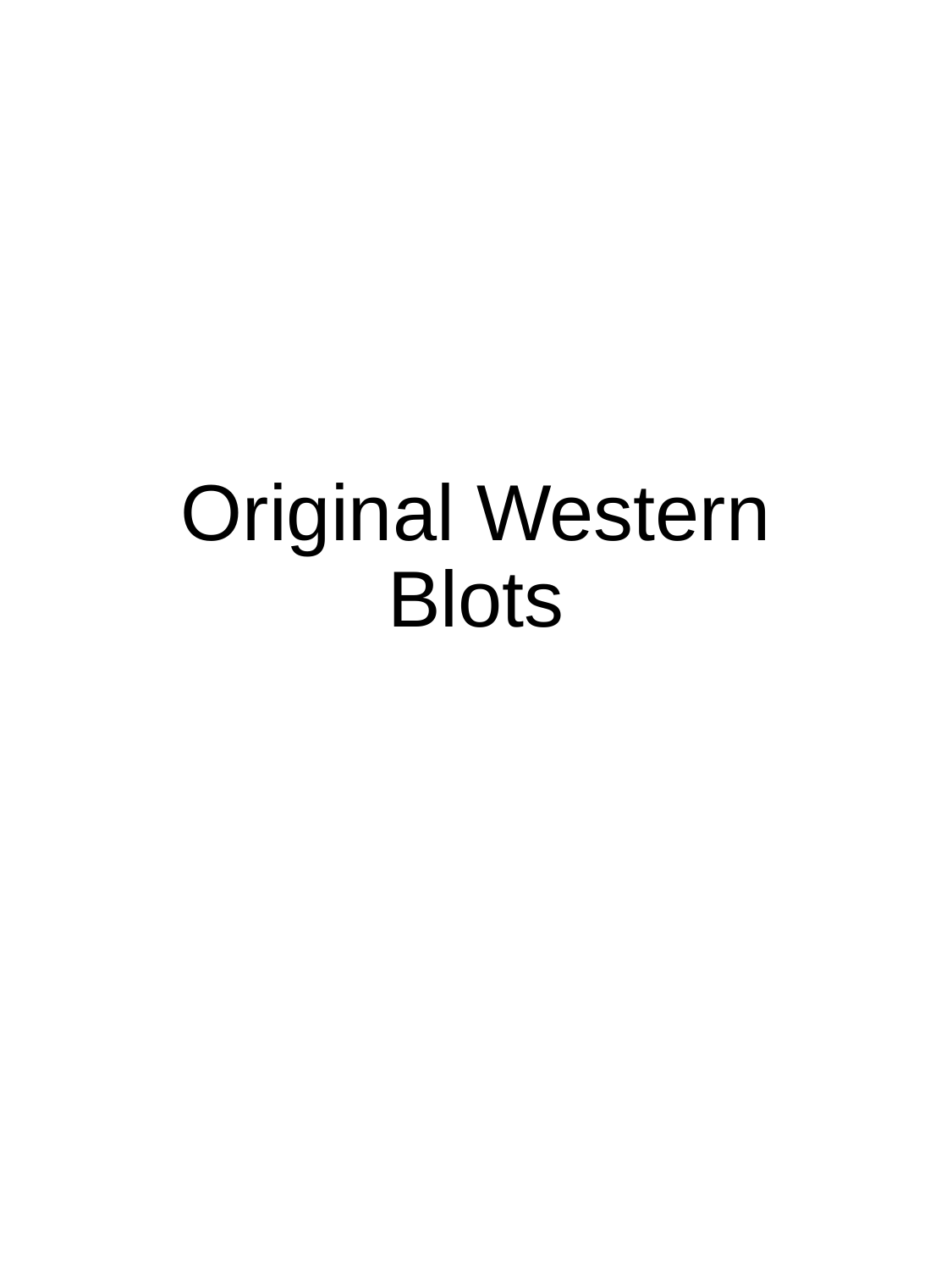

# Original Western Blots

## Slide 2
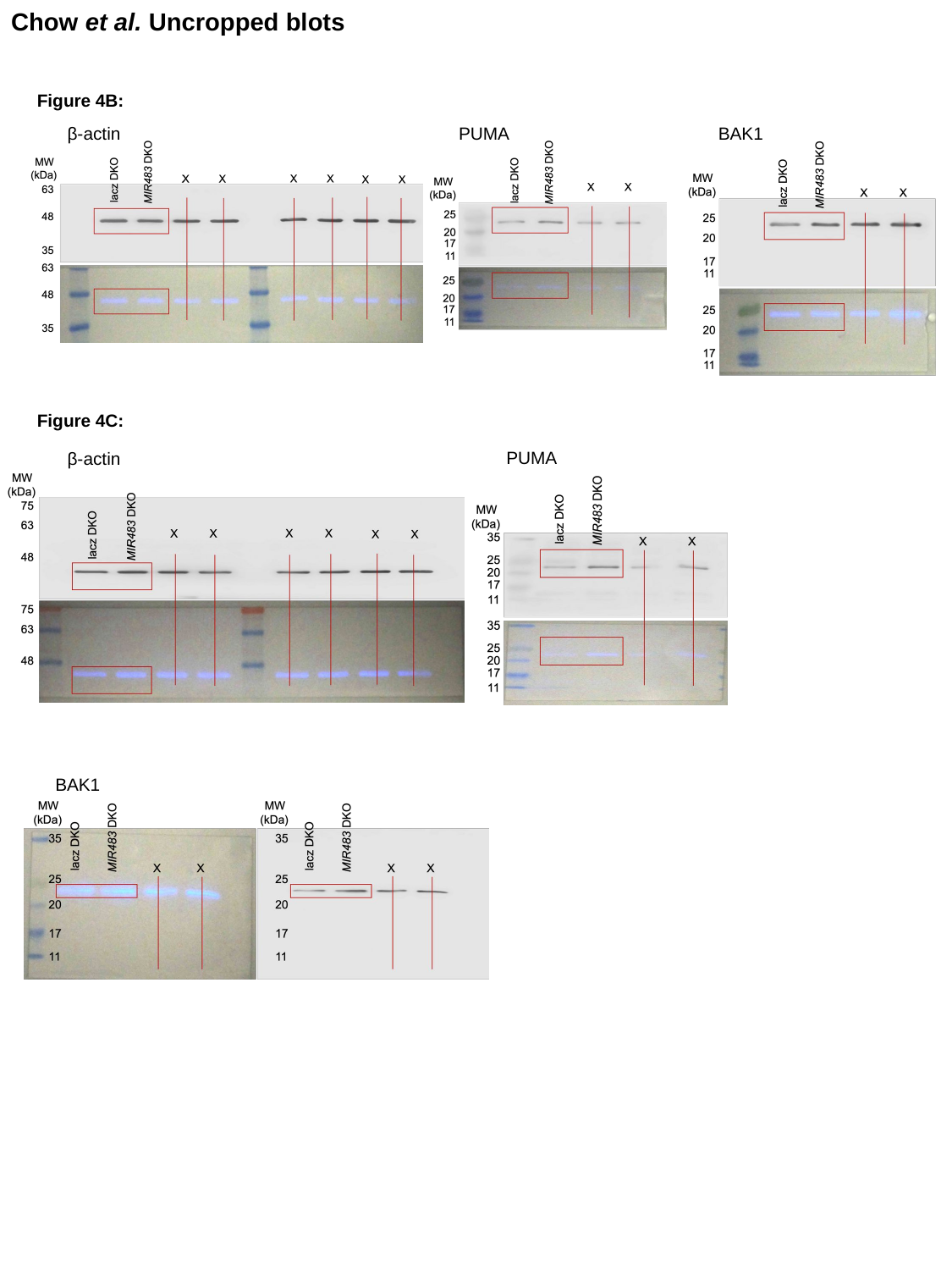

Chow et al. Uncropped blots
Figure 4B:
BAK1
PUMA
β-actin
Figure 4C:
PUMA
β-actin
BAK1

## Slide 3
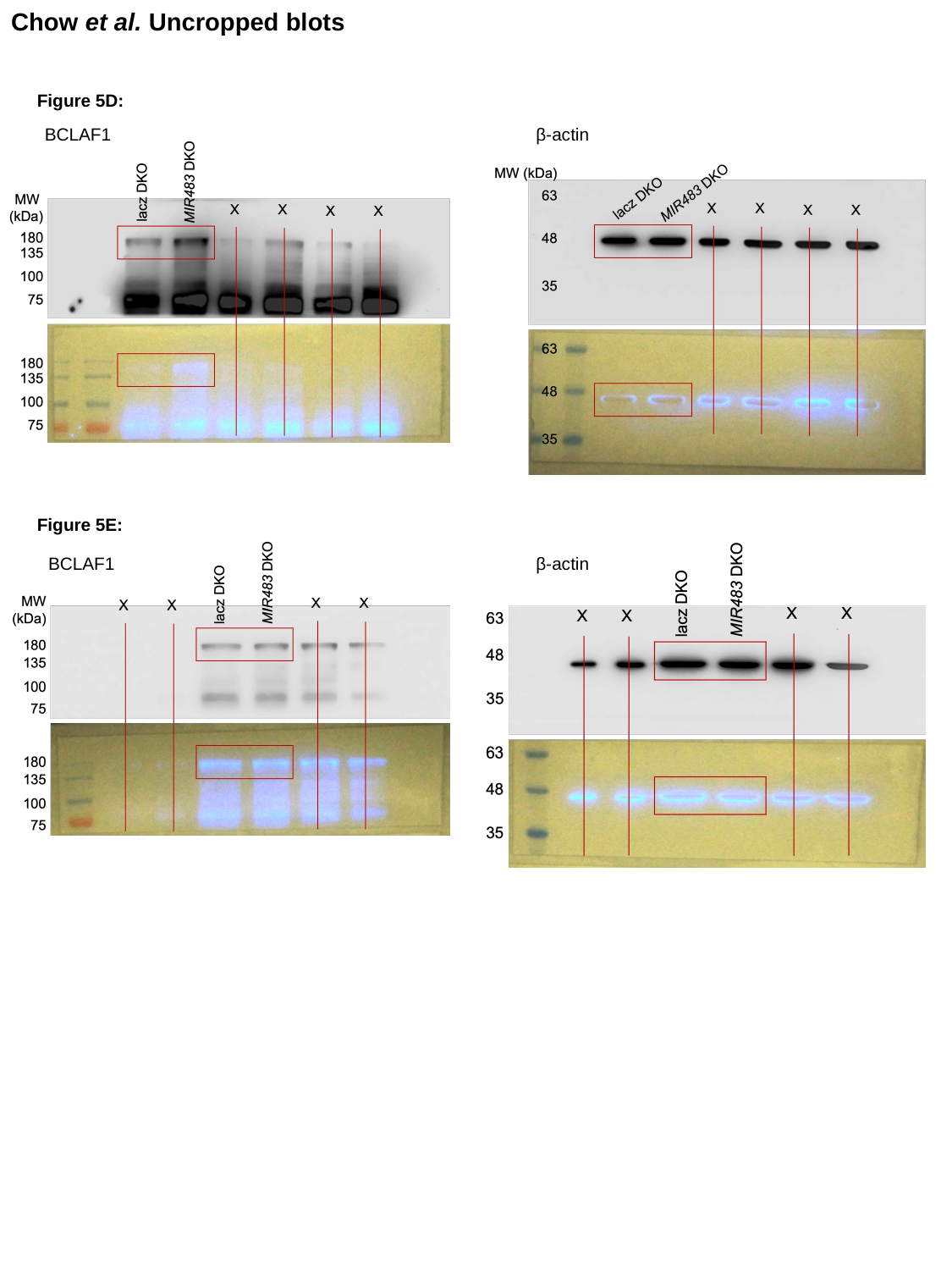

Chow et al. Uncropped blots
Figure 5D:
β-actin
BCLAF1
Figure 5E:
BCLAF1
β-actin

## Slide 4
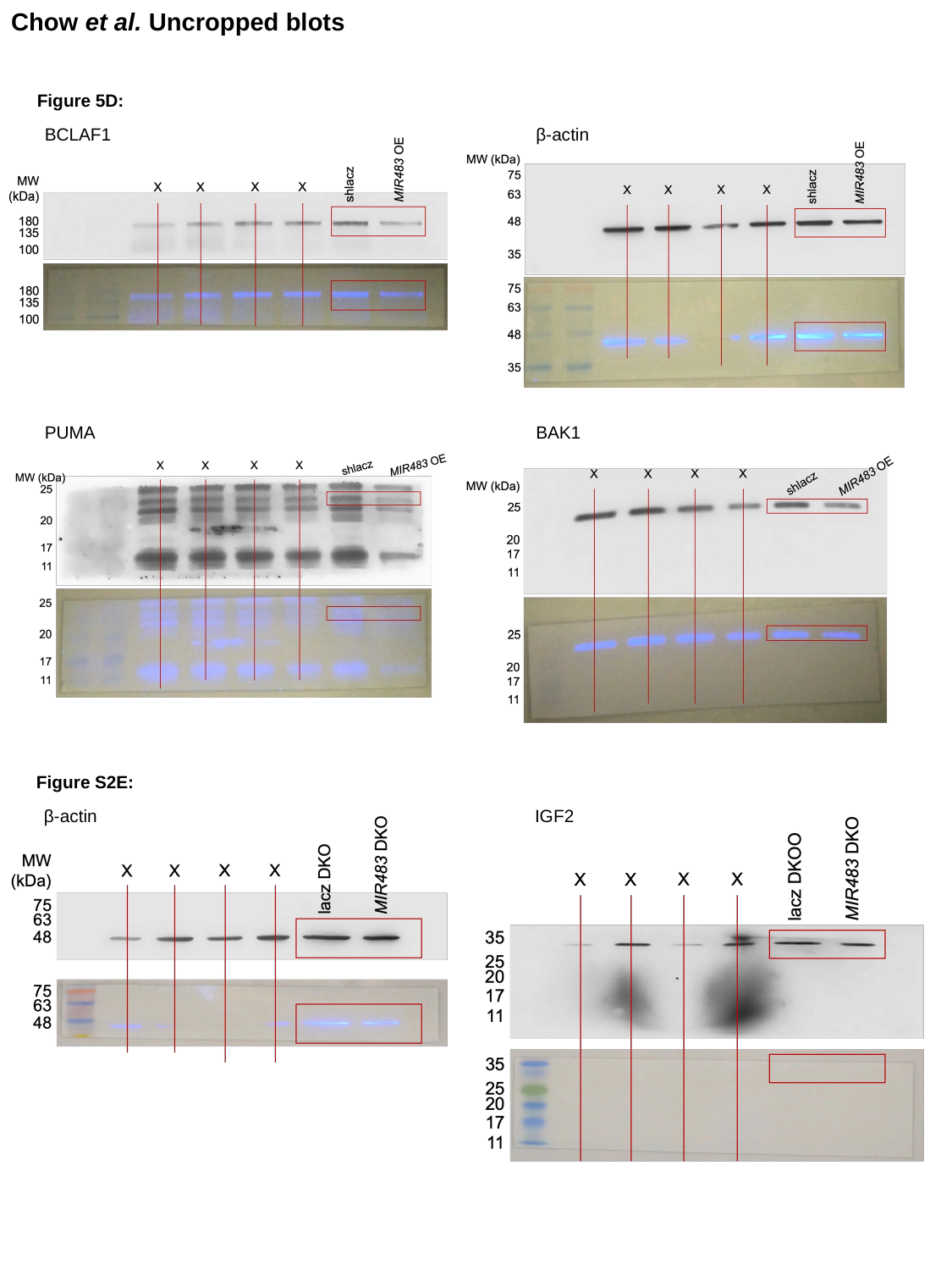

Chow et al. Uncropped blots
Figure 5D:
β-actin
BCLAF1
BAK1
PUMA
Figure S2E:
IGF2
β-actin

## Slide 5
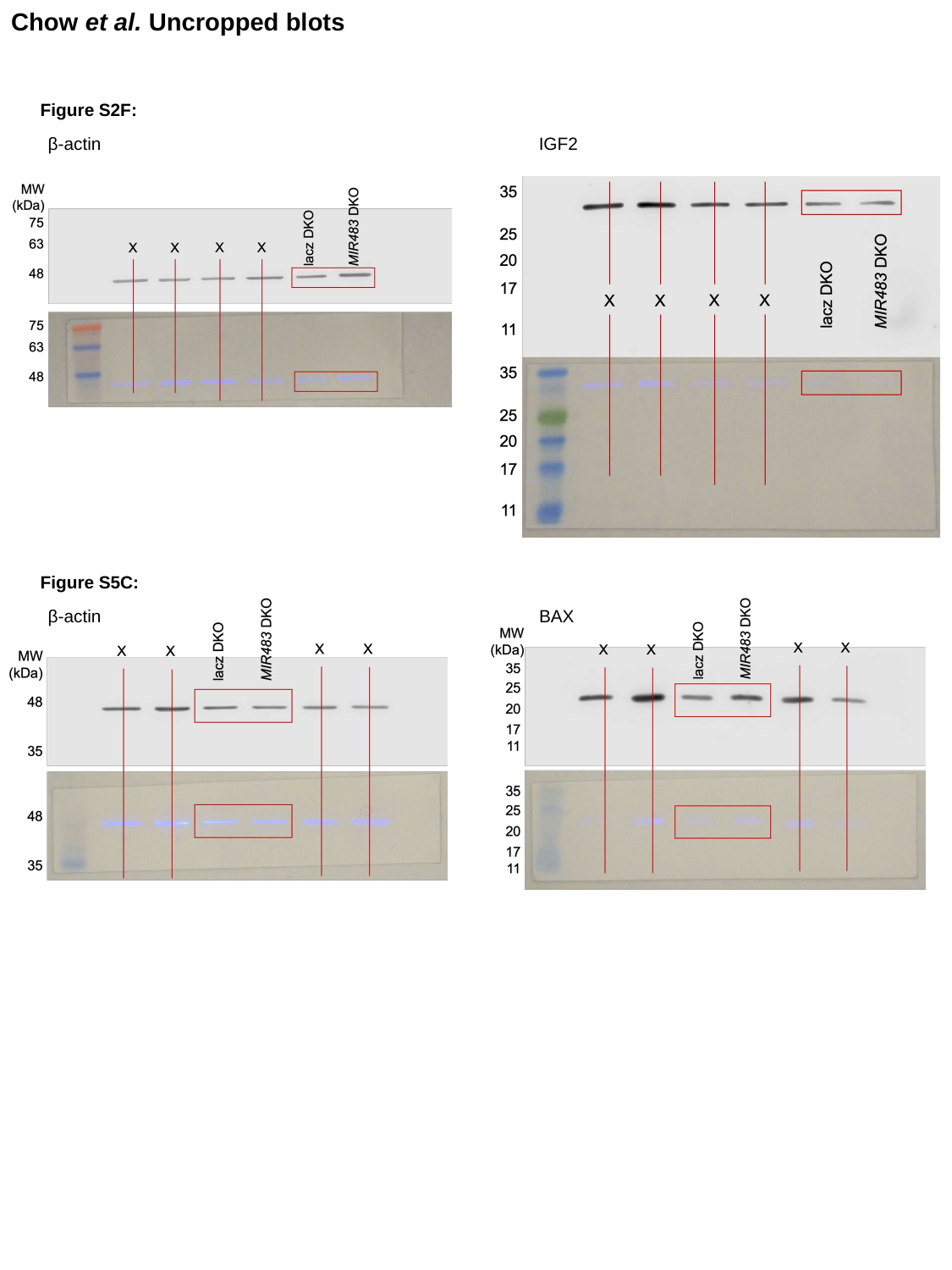

Chow et al. Uncropped blots
Figure S2F:
IGF2
β-actin
Figure S5C:
BAX
β-actin

## Slide 6
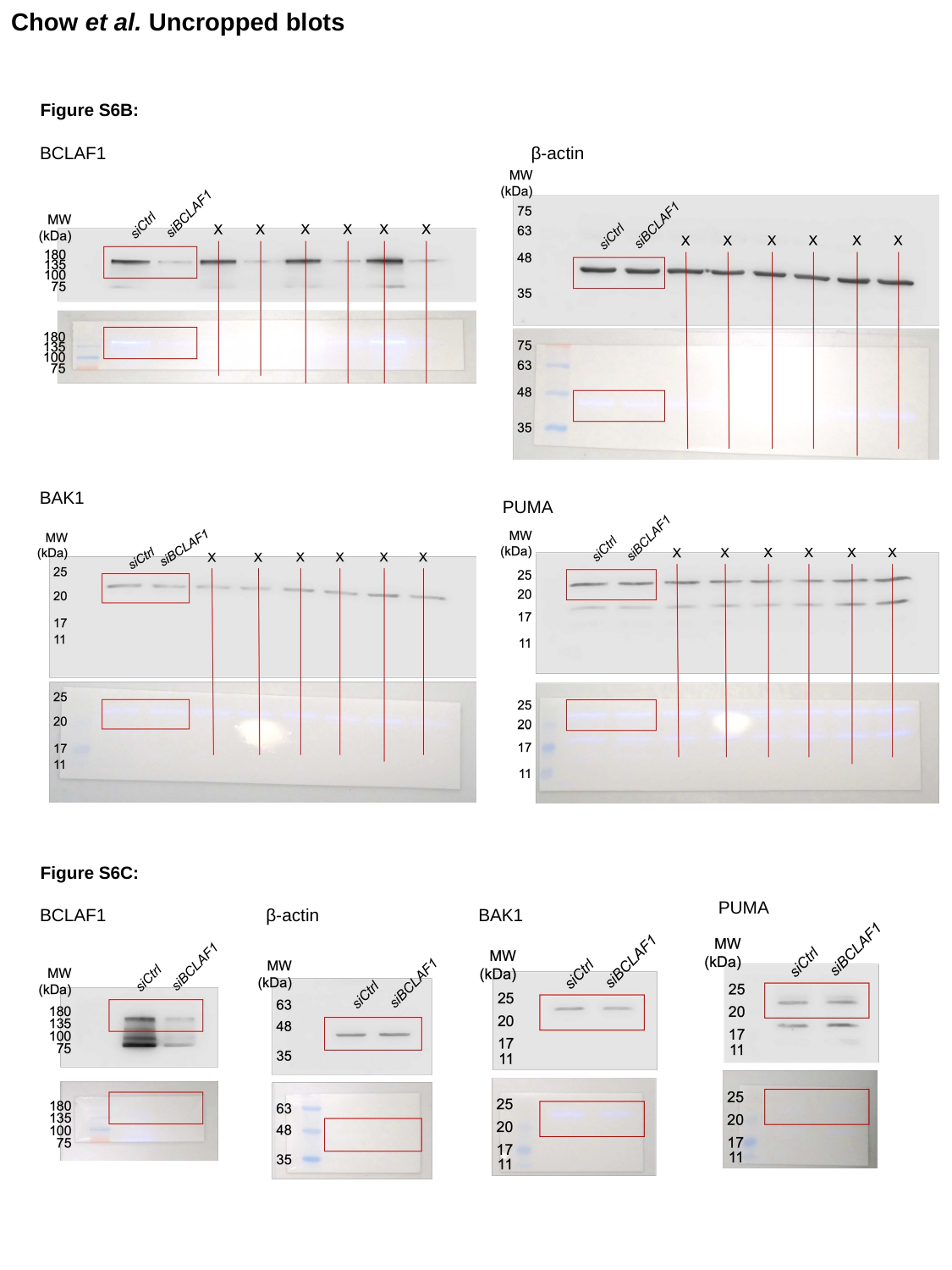

Chow et al. Uncropped blots
Figure S6B:
β-actin
BCLAF1
BAK1
PUMA
Figure S6C:
PUMA
BCLAF1
β-actin
BAK1
